# Plasma Cell Alloantigen 1 and IL-10 Secretion Define Two Distinct Peritoneal B1a B Cell Subsets With Opposite Functions, PC1^high^ Cells Being Protective and PC1^low^ Cells Harmful for the Growing Fetus

**DOI:** 10.3389/fimmu.2018.01045

**Published:** 2018-05-15

**Authors:** Anne Schumacher, Stefanie Ehrentraut, Markus Scharm, Hongsheng Wang, Roland Hartig, Herbert C. Morse, Ana Claudia Zenclussen

**Affiliations:** ^1^Department of Experimental Obstetrics and Gynecology, Medical Faculty, Otto-von-Guericke University, Magdeburg, Germany; ^2^Virology and Cellular Immunology Section, Laboratory of Immunogenetics, National Institute of Allergy and Infectious Diseases, NIH, Rockville, MD, United States; ^3^Core Facility Multidimensional Microscopy and Cellular Diagnostics, Medical Faculty, Otto-von-Guericke University, Magdeburg, Germany

**Keywords:** B cells, B1a B cells, fetal tolerance, interleukin-10, peritoneum, plasma cell alloantigen 1, pregnancy

## Abstract

B cells possess various immuno regulatory functions. However, research about their participation in tolerance induction toward the fetus is just emerging. Accumulating evidence supports the idea that B cells can play seemingly conflicting roles during pregnancy, either protecting or harming the fetus. Previous findings indicated the presence of two different peritoneal B cell subsets, defined by the expression of the plasma cell alloantigen 1 (PC1) and with distinct immune modulatory functions. Here, we aimed to study the participation of these two B cell subsets, on pregnancy outcome in a murine model of disturbed fetal tolerance. The frequencies and cell numbers of peritoneal and splenic CD19^+^IL-10^+^ and CD19^+^CD5^+^IL-10^+^PC1^+^ cells were assessed in virgin as well as normal pregnant (NP) and abortion-prone (AP) females during the course of gestation. Peritoneal PC1^low^ or PC1^high^ B1a B cells were sorted, analyzed for their ability to secrete IL-10 and adoptively transferred into NP or AP females. On gestation day (gd) 12, the abortion rate as well as the frequencies and cell numbers of regulatory T cells, TH1 and TH17 cells were determined in spleens and decidua. In addition, mRNA expression of IL-10, TGF-β, IFN-γ, and TNF-α was analyzed in decidual tissue. Peritoneal CD19^+^IL-10^+^ and CD19^+^CD5^+^IL-10^+^PC1^+^ frequencies fluctuated during the progression of normal pregnancies while no significant changes were observed in spleen. AP females showed significantly reduced frequencies of both B cell populations and exhibited an altered peritoneal PC1^high^/PC1^low^ ratio at gd10. Adoptive transfers of PC1^low^ B1a B cells into NP females increased the abortion rate in association with a reduced splenic regulatory T/TH17 ratio. By contrast, the transfer of PC1^high^ B1a B cells into AP females significantly diminished the fetal rejection rate and significantly reduced the numbers of splenic TH17 cells. Our results suggest that the peritoneum harbors two distinct B1a B cell subsets that can be distinguished by their PC1 expression. Whereas PC1^high^ B1a B cells seem to support fetal survival, PC1^low^ cells B1a B cells may compromise fetal well-being.

## Introduction

Subsets of B cells are capable of a wide range of functions directing them toward immune activation or tolerance. Studies designed to understand their possible contributions to immune regulation during pregnancy have indicated an involvement of B cells in both, pregnancy well-being and pregnancy complications (1). B1a B cells in particular seem to play an ambivalent role in pregnancy (1). This B cell population represents one of the three major B cell subtypes and can be distinguished from B1b B cells and from conventional B2 B cells by their developmental origins, surface marker expression, and functions (2). In mice, the majority of B1a B cells are found in the peritoneal cavity; however, they can also be found in pleural cavities and various parts of the intestine, albeit in lower numbers (3). B1a B cells are long-lived and possess a reduced B cell receptor diversity and affinity (4). They are largely responsible for the secretion of natural antibodies that are produced in the absence of antigenic stimuli and are characterized by their low affinity as well as poly- and self-reactivity (2). Based on these understandings, we proposed that human B cells functionally comparable to mouse CD19^+^CD5^+^ B1a B cells may be a source of poly-reactive autoantibodies that have been associated with the pregnancy disorder, preeclampsia (5). We showed that during the third trimester, the frequencies of CD19^+^CD5^+^ B1a B cells in the peripheral blood of preeclamptic patients are significantly increased compared with those of women with normal pregnancies (5). In addition, the increase in detrimental B1a B cells seems to be driven by pathologically elevated levels of the otherwise tolerogenic pregnancy hormone, human chorionic gonadotropin (5). Interestingly, normal levels of the same hormone can induce the generation of IL-10-producing regulatory B (Breg) cells (6) that are crucial for fetal tolerance induction in human pregnancies (7). In mice, regulatory B10 cells, defined by their ability to secrete IL-10, are augmented in normal pregnant (NP) females and do not increase in spontaneous abortion-prone (AP) females (8). Important, the pregnancy-protective B10 cells belong to the B1a B cell population (8). By contrast, in the same animal model, we found that B1a B cells from AP females but not from NP females induce TH1 and TH17 cells (9). We postulated that the differential function of B1a B cells during pregnancy might be associated with expression levels of the co-stimulatory molecule, CD86 (9). However, the intrinsic pathways driving B1a B cells to adopt tolerogenic or immunogenic phenotypes are far from understood. Wang and colleagues recently described two distinct B1a B cell subsets located in the peritoneal cavity based on differential expression of the protein initially termed plasma cell alloantigen 1 (PC1), encoded by the gene ectonucleotide pyrophosphatase phosphodiesterase 1 (*Enpp1*) (10). These studies were performed using non-pregnant animals (10). The authors designated the B1a B cell subsets as B-1a.PC1^hi^ and B-1a.PC1^lo^ and clearly identified differences in their ability to secrete IgM antibodies and IL-10 as well as in their capacity to induce TH1 differentiation (10). Even more interestingly, the levels of PC1 expression directly correlated with the ability of the two B1a B cell subsets to secrete IL-10. Based on their findings, the authors proposed that B-1a.PC1^hi^ cells possess tolerogenic properties while B-1a.PC1^lo^ cells behave rather immunogenic (10).

The aim of this study was to investigate whether PC1-expressing B1a B cells change in number during the progression of normal pregnancies and whether there are differences between NP and AP females. Moreover, we were eager to learn whether either B1a B cell subset might affect pregnancy outcome and, if so, the underlying mechanisms of action.

## Materials and Methods

### Animals

CBA/J female mice as well as BALB/c and DBA/2J male mice were purchased from Janvier Labs (Saint-Berthevin, France). All animals were maintained in our animal facility and handled according to the institutional guidelines with the ministerial approval (Landesverwaltungsamt Sachsen-Anhalt (AZ42502-2-868 UNIMD)). The experiments were conducted by authorized persons according to the Guide for care and use of animals in Agriculture research and teaching. Mice were kept under a 12 h light/12 h dark cycle and received water and food *ad libitum*. CBA/J female mice were mated either to BALB/c male mice (NP combination) or to DBA/2J male mice (spontaneous AP combination) representing a well-established AP model. Whereas NP females show a fetal rejection rate of 0–10% (median), AP females show a spontaneous fetal rejection rate of 20–50% (median) depending on the laboratory conditions. The increased fetal rejection rate in AP females is caused by a disturbed immunological tolerance to the paternal minor histocompatibility antigens (11). Paired females were checked twice a day for a vaginal plug whose appearance indicated day 0 of pregnancy. In the first set of experiments, pregnant females sacrificed at gestation days (gds) 0, 2, 5, 10, or 14 as well as virgin females were included for B cell analyses. In the second set of experiments, NP or AP females received adoptive B cell transfers after implantation that occurs at gd5, and were sacrificed on day 12 of gestation for determination of the abortion rate and for tissue processing.

### Fluorescence-Activated Cell Sorting and Adoptive Transfer of Sorted PC1^high^ or PC1^low^ B1a B Cells

Peritoneal washout cells were obtained from BALB/c-mated CBA/J NP females (gd13 or 14). CD19^+^CD5^+^PC1^high^ or CD19^+^CD5^+^PC1^low^ B1a B cells were purified by flow cytometry (FC) following staining for CD19, CD5, and PC1. After sorting, cells were washed once in PBS, centrifuged and resuspended in PBS. NP females were injected intraperitoneally (i.p.) with 1 × 10^5^ PC1^low^ B1a B cells, while AP females received 1 × 10^5^ PC1^high^ B1a B cells i.p. Injections (total volume of 200 µl) were performed between gd7 and 9. Females injected with 200 µl PBS served as controls. As the number of PC1^high^ B1a B cells that could be obtained from a single pregnant donor mouse did not reach the required number of 1 × 10^5^ cells for adoptive transfer, we pooled PC1^high^ B1a B cells from 3 to 4 pregnant female donors.

### Cell Culture of Sorted PC1^high^ or PC1^low^ B1a B Cells and IL-10 Determination *via* Enzyme-Linked Immunosorbent Assay (ELISA)

After sorting, cells were washed once in RPMI 1640 medium (Thermo Fisher Scientific, Germany) supplemented with 10% fetal bovine serum (Merck Millipore, Germany) and 100 nM penicillin/streptomycin (Thermo Fisher Scientific, Germany). Then, 1 × 10^4^ PC1^high^ or PC1^low^ B1a B cells were cultured for 24 h in medium supplemented with 10 µg/ml lipopolysaccharide (LPS, Sigma, Germany) and 25 ng/ml Phorbol 12-myristate 13-acetate (PMA, Sigma, Germany). Supernatants were harvested and analyzed for IL-10 levels by ELISA using the BD OptEIA™ Mouse IL-10 ELISA Set (BD Biosciences, Germany) according to the manufacturer’s instructions.

### Tissue Sampling, Isolation of Mononuclear Cells, and Cell Stimulation

Virgin as well as pregnant females (gd0, 2, 5, 10, 12, or 14) were sacrificed by cervical dislocation. The spleen was removed and used to prepare single cell suspensions. Peritoneal cells were obtained by peritoneal lavage. For this purpose, a 30 ml syringe was filled with 10 ml Hanks’ Balanced Salt Solution (Sigma, Germany) and 2 ml air. The air–fluid mixture was injected into the peritoneum of the anesthetized female and equally dispersed by carefully shaking the animal for 3–4 min. Afterward, the cell suspension (7–8 ml) containing total peritoneal cells was sucked out of the peritoneal cavity using another syringe and transferred into a reaction tube. Mononuclear cells from the spleen and the peritoneum were further isolated using our established protocol (11). Briefly, splenic tissue was disaggregated and filtered through a sterile 100 µm net (BD Biosciences, Germany) using RPMI 1640 medium. Afterward, erythrocytes within splenic and peritoneal cell suspensions were lysed with an NHCl_4_/NaCl solution. Following centrifugation, splenic and peritoneal cells were washed in RPMI 1640 medium. 2 × 10^6^ spleen and peritoneal cells were stimulated for 4 h medium supplemented with 50 ng/ml PMA, 1 µg/ml ionomycin (Thermo Fisher Scientific, Germany), and 10 µg/ml LPS (Sigma, Germany). After 1 h of stimulation, 2 µM monensin (Sigma, Germany) was additionally introduced to the cultures.

To determine the number of implantations and the abortion rate, the pregnant uteri were opened longitudinally. The percentage of abortions was calculated as the ratio of resorption sites to total implantation sites (resorption plus normal implantation sites) multiplied by 100.

Fetoplacental units were separated from their implantation sites and a piece of decidua was snap frozen for real-time RT-PCR analysis. The remaining decidua was cut into small pieces and mononuclear cells were isolated according to our established protocol (12).

### FC Analysis

Stimulated mononuclear cells obtained from the spleen, peritoneum, and decidua of virgin, and pregnant females were stained for B and T cell markers as described elsewhere (12). Briefly, cells were washed in PBS containing 1% bovine serum albumin (BSA) and 0.1% sodium azide (FC buffer). Thereby, the amount of BSA added to the buffer is sufficient to block unspecific staining of Fc receptors. Afterward, staining for extracellular markers (1:100 antibody dilutions) was performed for 30 min at 4°C in the dark. Following another washing step in FC buffer, cells were fixed overnight using the fixation/permeabilization set from Thermo Fisher Scientific, Germany. For intracellular staining, cells were washed in permeabilization buffer and then incubated for 30 min at 4°C in the dark in the staining solution (1:200 antibody dilutions). After washing in permeabilization buffer, cells were resuspended in FC buffer and measured using a 4-color FACSCalibur flow cytometer from Becton Dickinson, Germany. Per sample, 5 × 10^4^ splenic and peritoneal cells as well as 5 × 10^3^ decidual cells were acquired. Percentages and cell numbers of B and T cells were determined within the acquired cell pool. Data analysis was performed using CellQuest Pro software (version: 0.3.5fbb; Becton Dickinson, Germany). Primary gates were set on either total mononuclear cells or lymphocytes in the FSC/SSC plots. Cell debris and dead cells were excluded from analysis by estimated cell size. Furthermore, to determine positive versus negative cells, fluorescence minus one (FMO) controls were applied. Individual gating strategies and representative dot plots are included in each figure.

The following antibodies were used: FITC-conjugated anti-mouse CD5 (clone: 53-7.3), PE-conjugated anti-mouse IL-10 (clone: JES5-16E3), PerCP-conjugated anti-mouse CD19 (clone: 6D5), FITC-conjugated anti-mouse CD4 (clone: RM4-4), PE-conjugated anti-mouse Foxp3 (clone: FJK-16s), PE-conjugated anti-mouse TNF-α (clone: MP6-XT22), PerCP-Cy5.5-conjugated anti-mouse IFN-γ (clone: XMG1.2), PE-conjugated anti-mouse IL-17 (clone: TC11-18H10), and APC-conjugated anti-mouse IL-10 (clone: JES5-16E3). The APC-conjugated PC1 (clone: YE1/19.1) was produced as described previously (12). Foxp3 antibody was purchased from eBioscience, Germany. All other antibodies were purchased from Becton Dickinson, Germany.

### Real-Time RT-PCR Analysis

Frozen decidual tissue was treated with Trizol^®^ Reagent (Thermo Fisher Scientific, Germany) and disaggregated using a homogenizer (Ultra-Turrax T8; IKA, Germany). RNA was extracted with chloroform, precipitated with 2-propanol, washed in 80% ethanol, and finally diluted in RNase-free water. The RNA was quantified by reading ultraviolet absorbance at 260 nm. For cDNA synthesis, 2 µg of total RNA were placed for 2 min on ice and added with dNTPs (2.5 mmol/l, Amersham Biosciences, Germany), DNase I (2 U/ml, Sigma, Germany), and RNase inhibitor (40 U/ml, Promega, Germany) mixed in reaction buffer. The mix was incubated for 30 min at 37°C and further heated to 75°C for 5 min. The addition of the reverse transcriptase (200 U/ml, Amersham Biosciences, Germany) and RNase inhibitor started the reverse transcription. This reaction mixture was incubated at 42°C for 1 h followed by incubation at 94°C for 5 min.

For detection of IL-10, TGF-β, IFN-γ, and TNF-α TaqMan technology was conducted using an iQ5 Multicolor RT-PCR Detection System (Bio-Rad Laboratories, Germany). The amplification reactions (12 µl) consisted of 1 µl of cDNA, 6.25 µl of mastermix containing PCR buffer, dNTPs, MgCl_2_, and Ampli-*Taq* DNA polymerase (Thermo Fisher Scientific, Germany), 3 µl of the primer mix, 1.25 µl of water, and 0.5 µl of the fluorescent probes. PCR reaction was performed as follows: 2 min at 50°C followed by an initial denaturation step of 10 min at 95°C, followed by 15 s at 95°C, and 1 min at the appropriate annealing temperature for 40 cycles. β-Actin was employed as housekeeping gene, and relative gene expression was calculated by 2^−ΔCT^. Primer and probe sequences (Metabion, Germany) were the following: IL-10 sense primer: GAA GAC CCT CAG GAT GCG G; IL-10 anti-sense primer: CCT GCT CCA CTG CCT TGC T; IL-10 probe: Fam-CGC TGT CAT CGA TTT CTC CCC TGT GA-Tamra; TGF-β sense primer: GGC TAC CAT GCC AAC TTC TGT CT; TGF-β anti-sense primer: CCG GGT TGT GTT GGT TGT AGA; TGF-β probe: Fam-CAC ACA GTA CAG CAA GGT CCT TGC CCT-Tamra; IFN-γ sense primer: AGC TCA TTG AAT GCT TGG CG; IFN-γ anti-sense primer: AGC AAC AGC AAG GCG AAA AA; IFN-γ probe: Fam-ATT GCC AAG TTT GAG GTC AAC AAC CCA CA-Tamra; TNF-α sense primer: TCG AGT GAC AAG CCC GTA GC; TNF-α anti-sense primer: CTC AGC CAC TCC AGC TGC TC; TNF-α probe: Fam-CGT CGT AGC AAA CCA CCA AGC GGA GGA-Tamra.

### Data Analysis and Statistics

Analysis of data was performed using the GraphPad Prism 7.01 software (STATCON, Germany). Data obtained from ELISA assays are presented as means + SEM and were analyzed using the Wilcoxon matched-pairs signed rank test. All other data sets are presented as medians in graphs showing individual values for each animal. Data analysis was performed using the non-parametric Kruskal–Wallis test, followed by the Mann–Whitney-*U* test. In all cases, *p* ≤ 0.05 was considered to be statistical significant.

## Results

### Reduced Numbers of Splenic and Peritoneal IL-10-Producing CD19^+^ B Cells in AP Females

**Figure 1:**
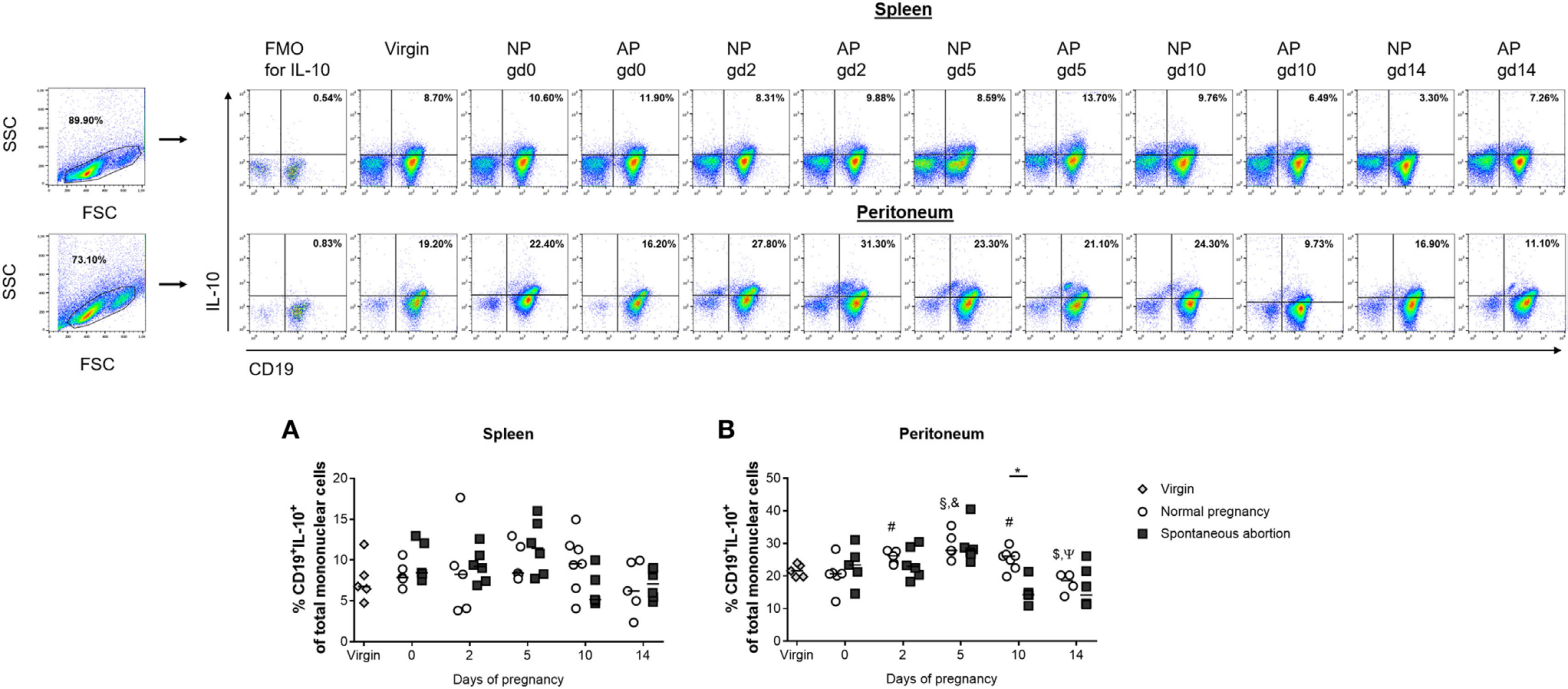
The frequencies of peritoneal CD19^+^IL-10^+^ B cells were significantly reduced in abortion-prone (AP) females on gestation day (gd) 10. Virgin (*n* = 5 per organ) as well as normal pregnant (NP, *n* = 4–7 per organ) and AP females (*n* = 5–6 per organ) were analyzed at gd0, 2, 5, 10, and 14 for the frequencies of CD19^+^IL-10^+^ B cells in spleen **(A)** and peritoneum **(B)**. Flow cytometry gating strategy and representative dot plots are included in this figure. Graphical data are displayed as medians. Each symbol represents a single animal, and the presented number of animals refers to pooled data of at least two independent experiments. Statistical analysis was performed using the non-parametric Kruskal–Wallis test followed by the Mann–Whitney-*U* test (*p ≤ 0.05; ^#^*p* ≤ 0.05 vs. virgin, ^§^*p* ≤ 0.01 vs. virgin, ^&^*p* ≤ 0.05 vs. day 0, ^$^*p* ≤ 0.05 vs. day 5, and ^Ψ^*p* ≤ 0.05 vs. day 10). Abbreviation: FMO, fluorescence minus one.

We previously suggested that IL-10-producing Breg cells are associated with fetal protection (8). Here, we first sought to determine the frequencies and cell numbers of total B cells with the capacity to produce IL-10 after *in vitro* stimulation of mononuclear cells obtained from the spleen and peritoneum of virgin and pregnant females. We found no significant alterations in the frequencies and cell numbers of splenic CD19^+^ B cells with the ability to produce IL-10 throughout normal pregnancies (Figure 1A; Figure S1A in Supplementary Material). By contrast, the frequencies and cell numbers of peritoneal CD19^+^ B cells with the capability to produce IL-10 increased significantly in the pre-implantation period, peaked around implantation and declined significantly thereafter (Figure 1B; Figure S1B in Supplementary Material). We also asked whether the frequencies and cell numbers of CD19^+^ B cells able to produce IL-10 after *in vitro* stimulation differed between NP and AP females using a well-established murine model of disturbed fetal tolerance (11). On gd10 we observed significant reductions in the cell numbers of CD19^+^IL-10^+^ B cells in spleens from AP as compared with NP females. Moreover, we detected significant reductions in the frequencies (gd10) and cell numbers (gd2 and 10) in peritoneum when comparing AP females with NP females (Figures 1A,B; Figures S1A,B in Supplementary Material). Further analysis revealed that all changes observed in the CD19^+^IL-10^+^ B cell population were not the result of changes in the total CD19^+^ B cell pool as no alterations in the numbers of CD19^+^ B cells could be found throughout pregnancy neither in NP females and in AP females nor between both pregnancy groups (data not shown). Moreover, determination of the frequencies of CD19^+^IL-10^+^ cells within the total CD19^+^ B cell population, indeed showed that particularly B cells with the ability to produce IL-10 increased throughout normal pregnancy in the peritoneum and were significantly elevated on gd10 in splenic and peritoneal tissue of NP females when compared with AP females (Table 1).

**Table 1:** The frequencies of splenic and peritoneal CD19^+^IL-10^+^ cells within total CD19^+^ B cells were significantly reduced in AP females on gd10.

CD19^**+**^IL-10^**+**^ within CD19^**+**^	Spleen					Peritoneum				
	gd0	gd2	gd5	gd10	gd14	gd0	gd2	gd5	gd10	gd14
Virgin	11.0					19.7				
NP	14.5	10.5	12.7	15.4	13.1	20.2	24.7	27.3	24.4	19.0
AP	10.1	13.1	17.2	9.7*	13.2	25.0	23.1	24.5^#^	11.7**	15.5

*The frequencies of CD19^+^IL-10^+^ B cells within total CD19^+^ B cells were determined in spleen and peritoneum from virgin (*n* = 5 per organ), NP (*n* = 4–7 per organ), and AP (*n* = 5–6 per organ) females at different gestation days (gds). For flow cytometry analysis, a first gate was set on all mononuclear cells, followed by a representation of CD19^+^ cells within this gate. Next, a second gate was set on all CD19^+^ cells, and within the second gate IL-10 was plotted against CD19. Data are displayed as medians*.

Statistical analysis was performed using the Mann–Whitney-*U* test (*p ≤ 0.05 vs NP; **p ≤ 0.01 vs NP; and ^#^*p* ≤ 0.05 vs virgin).

*NP, normal pregnant; AP, abortion prone*.

### AP Females Had Significantly Reduced Numbers of Splenic and Peritoneal IL-10- and PC1-Coexpressing B1a B Cells and a Reduced PC1^high^/PC1^low^ B1a B Cell Ratio on gd10

**Figure 2:**
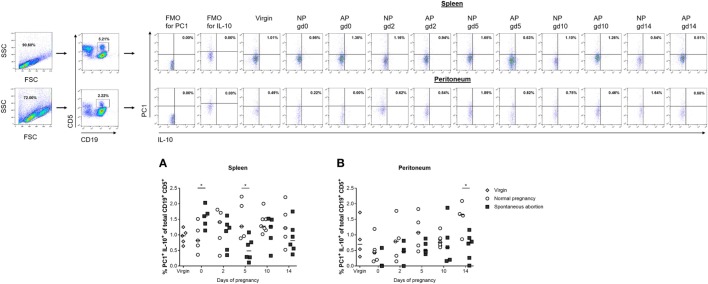
The frequencies of peritoneal CD19^+^CD5^+^PC1^+^IL-10^+^ B cells were significantly reduced in abortion-prone (AP) females on gestation day (gd) 14. Virgin (*n* = 4–5 per organ) as well as normal pregnant (NP, *n* = 4–7 per organ) and AP females (*n* = 5–6 per organ) were analyzed at gd0, 2, 5, 10, and 14 for the frequencies of PC1^+^IL-10^+^ cells within the CD19^+^CD5^+^ B1a B cell population in spleen **(A)** and peritoneum **(B)**. Flow cytometry gating strategy and representative dot plots are included in this figure. Graphical data are displayed as medians. Each symbol represents a single animal, and the presented number of animals refers to pooled data of at least two independent experiments. Statistical analysis was performed using the non-parametric Kruskal–Wallis test followed by the Mann–Whitney-*U* test (*p ≤ 0.05). Abbreviation: FMO, fluorescence minus one.

Next, we asked if the observed alterations in IL-10-expressing cells in the total CD19^+^ B cell pool was reflected within the CD19^+^CD5^+^ B1a B cell subpopulation and whether these cells might additionally express PC1. We studied the distributions of IL-10- and PC1-coexpressing CD19^+^CD5^+^ B1a B cells in the spleen and peritoneum of NP females compared with AP females. In NP females, we did not detect significant changes at different gds in either tissue sites (Figures 2A,B; Figures S1C,D in Supplementary Material). Comparisons between AP and NP females revealed significant reduced frequencies of IL-10- and PC1-coexpressing CD19^+^CD5^+^ B1a B cells in spleens on gd5 (Figure 2A) as well as in the frequencies and cell numbers on gd0 and 14 in peritoneal cavities (Figure 2B; Figure S1D in Supplementary Material).

Furthermore, we asked whether the ratio of PC1^high^ to PC1^low^ B1a B cells is altered in pathologic pregnancies when compared with normal pregnancies. We found a significantly diminished PC1^high^/PC1^low^ ratio after implantation at gd10 in peritoneal cavities, whereas no statistically significant alterations could be detected in spleen at all time points (Tables 2 and 3). Further analysis revealed that the reduced peritoneal PC1^high^/PC1^low^ ratio (gd10) is provoked by a decrease in the number of PC1^high^ cells in AP females (data not shown). Compared with virgin females, a significant reduction in the PC1^high^/PC1^low^ ratio in splenic and peritoneal tissues of pregnant females from both pregnancy groups could be observed on gd10 (Tables 2 and 3).

**Table 2:** The PC1^high^/PC1^low^ ratio was significantly reduced in peritoneum from AP females on gd10.

PC1^high^/PC1^low^	Spleen					Peritoneum				
	gd0	gd2	gd5	gd10	gd14	gd0	gd2	gd5	gd10	gd14
Virgin	0.56					2.04				
NP	0.21	0.24	0.16	0.27	0.55	0.41	0.64	0.49	0.66	0.55
AP	0.16	0.21	0.17	0.13^#^	0.49	0.37	0.61	0.30	0.11*^,^^ψ^	0.44

*The frequencies of CD19^+^CD5^+^PC1^high^ and CD19^+^CD5^+^PC1^low^ were determined in spleen and peritoneum from virgin (*n* = 5 per organ), NP (*n* = 4–7 per organ), and AP (*n* = 5–6 per organ) females at different gestation days (gds), and the ratio between both B1a B cell subpopulations was calculated. Data are displayed as medians*.

Statistical analysis was performed using the Mann–Whitney-*U* test (*p ≤ 0.05 vs NP; ^#^*p* ≤ 0.05 vs virgin; and ^Ψ^*p* ≤ 0.01 vs virgin).

*NP, normal pregnant; AP, abortion prone*.

**Table 3:** The PC1^high^/PC1^low^ ratio was significantly reduced in peritoneum from AP females on gd10.

PC1^high^/PC1^low^	Spleen					Peritoneum				
	gd0	gd2	gd5	gd10	gd14	gd0	gd2	gd5	gd10	gd14
Virgin	0.56					1.17				
NP	0.21	0.24	0.15	0.27	0.55	0.15	0.25	0.35	0.29	0.55
AP	0.16	0.22	0.17	0.13^#^	0.47	0.14	0.23	0.11	0.11*	0.16

*The cell numbers of CD19^+^CD5^+^PC1^high^ and CD19^+^CD5^+^PC1^low^ were determined in spleen and peritoneum from virgin (*n* = 5 per organ), NP (*n* = 4–7 per organ), and AP (*n* = 5–6 per organ) females at different gestation days (gds), and the ratio between both B1a B cell subpopulations was calculated. Data are displayed as medians*.

Statistical analysis was performed using the Mann–Whitney-*U* test (*p ≤ 0.05 vs NP and ^#^*p* ≤ 0.05 vs virgin).

*NP, normal pregnant; AP, abortion prone*.

### Peritoneal PC1^high^ Cells From NP Females Expressed Significantly More IL-10 Than Their PC1^low^ Counterparts

**Figure 3:**
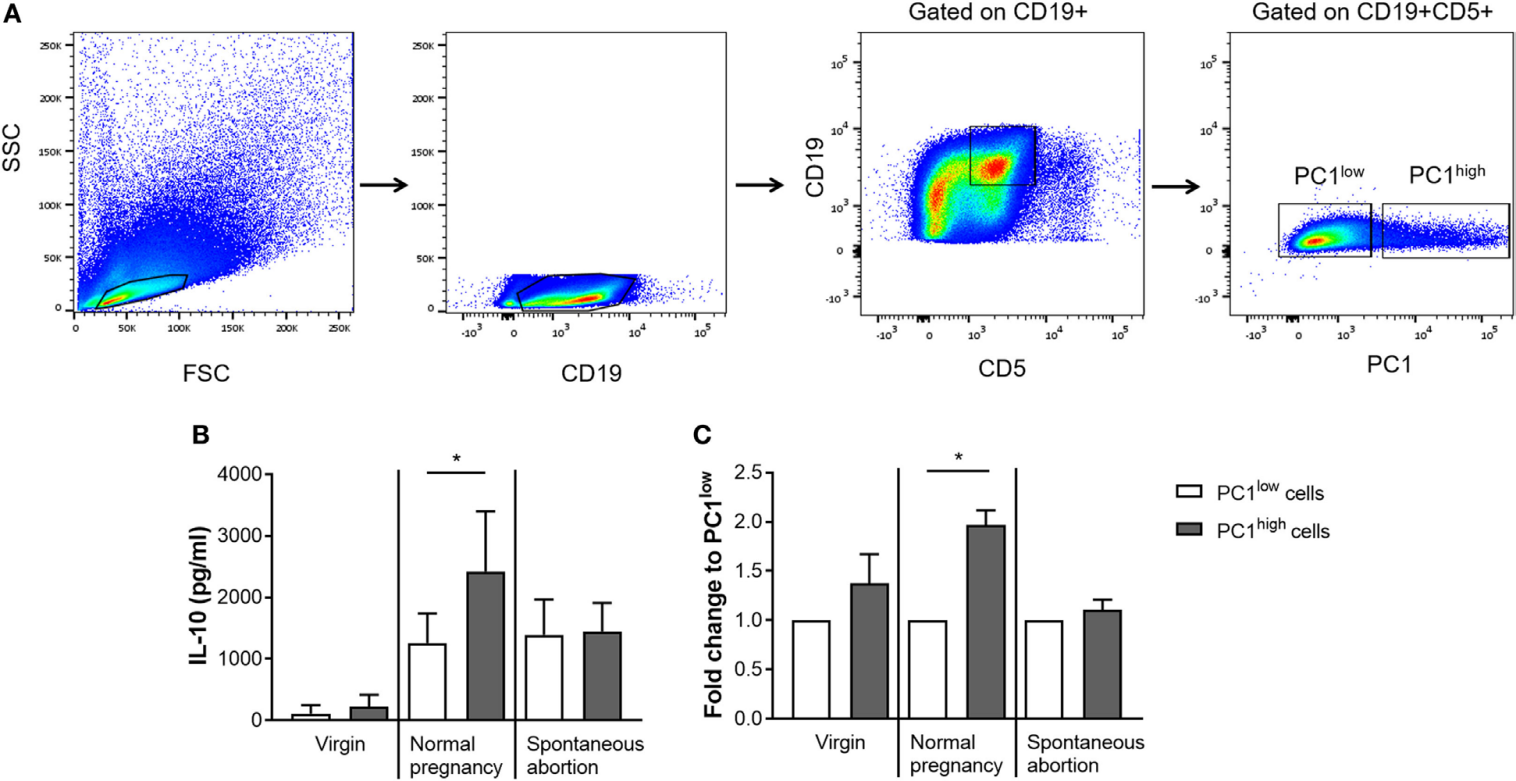
Peritoneal PC1^high^ cells from normal pregnant (NP) females secreted significantly more IL-10 than their PC1^low^ counterparts obtained from the same animals. Peritoneal CD19^+^CD5^+^ B1a B cells were sorted from virgin (*n* = 2), NP (*n* = 6), and abortion-prone (*n* = 3) females based on the height of their PC1 expression and subdivided in PC1^low^ and PC1^high^ cells. The sorting strategy is displayed in panel **(A)**. Sorted cells were cultured for 24 h in medium containing lipopolysaccharide and phorbol 12-myristate 13-acetate. Afterward, the levels of IL-10 secreted by either PC1^low^ or PC1^high^ cells were determined **(B,C)**. Data are displayed as means + SEM. Analysis was performed by using the Wilcoxon matched-pairs signed rank test (*p ≤ 0.05).

After we confirmed in pregnant animals that IL-10-expressing B1a B cells can coexpress PC1, we asked whether there is a correlation in the expression pattern between both molecules. Moreover, as differences in the frequencies of IL-10- and PC1-coexpressing B1a B cells as well as in the PC1^high^/PC1^low^ ratio between AP and NP females were more pronounced in the peritoneum, we decided to sort for PC1^high^ and PC1^low^ B1a B cells from the peritoneal cavity based on the sorting strategy displayed in Figure 3A. After sorting, the cells were stimulated with LPS and PMA for 24 h and the amount of secreted IL-10 in the supernatants was quantified. In agreement with the study by Wang and colleagues (10), we found PC1^high^ cells from virgin females secreted more IL-10 than PC1^low^ cells (Figures 3B,C). Moreover, PC1^high^ cells obtained from NP females secreted significantly higher amounts of IL-10 than their PC1^low^ counterparts. This was not the case for PC1^high^ cells obtained from AP females (Figures 3B,C). In addition, PC1^high^ cells from NP females had a greater capacity to secrete IL-10 than PC1^high^ cells from virgin and AP females (Figure 3B).

### Adoptive Transfer of PC1^low^ B1a B Cells Into NP Females Provoked Fetal Rejection

**Figure 4:**
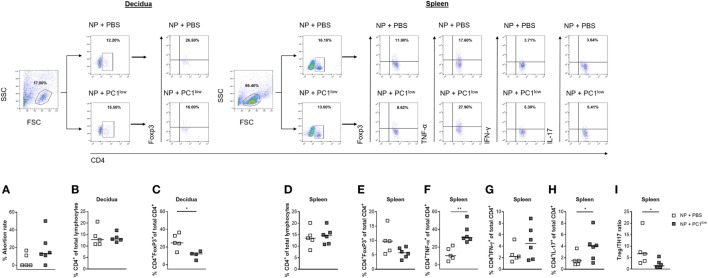
Adoptive transfer of PC1^low^ B1a B cells to NP females resulted in an increased fetal rejection rate and significantly reduced the splenic regulatory T (Treg)/TH17 ratio. 1 × 10^5^ peritoneal sort-purified PC1^low^ B1a B cells from NP females [gestation days (gds) 13–14] were adoptively transferred into NP females (*n* = 6) on gd7–9. Control females (*n* = 5) received PBS. The recipients were sacrificed on gd12, and the abortion rate **(A)** was determined. Moreover, CD4^+^ T cell **(B)** and CD4^+^Foxp3^+^ Treg frequencies **(C)** were assessed in decidual tissue. In spleen, the frequencies of CD4^+^ T cells **(D)**, CD4^+^Foxp3^+^ Treg cells **(E)**, CD4^+^TNF-α^+^ **(F)**, and CD4^+^IFN-γ^+^ **(G)** TH1 cells as well as the frequencies of CD4^+^IL-17^+^ TH17 **(H)** cells and the Treg/Th17 ratio **(I)** were determined. Flow cytometry gating strategy and representative dot plots are included in this figure. Graphical data are displayed as medians. Each symbol represents a single animal, and the presented number of mice refers to pooled data of two independent experiments. Statistical analysis was performed using the non-parametric Mann–Whitney-*U* test (*p ≤ 0.05 and **p ≤ 0.01). Abbreviation: NP, normal pregnant.

Based on our findings that AP females had a disturbed PC1^high^/PC1^low^ ratio, we hypothesized that the balance of PC1^high^ to PC1^low^ B1a B cells in the peritoneum may influence pregnancy outcome. To test our hypothesis, we first adoptively transferred sorted PC1^low^ B1a B cells i.p. after implantation and observed that the transfer of these cells resulted in an increased fetal rejection rate in otherwise NP females (Figure 4A).

### Adoptive Transfer of PC1^low^ B1a B Cells to NP Females Significantly Reduced the Splenic Regulatory T (Treg)/TH17 Ratio

Next, we asked if the detrimental effect of PC1^low^ B1a B cells on the success of pregnancy could be explained by alterations in the number of TH cell populations. We therefore determined the frequencies and cell numbers of CD4^+^ T cells, Treg cells, TH1 cells, and TH17 cells in the periphery as well as at the fetal–maternal interface. While the frequencies and cell numbers of CD4^+^ T cells were not altered in the decidua (Figure 4B; Figure S2A in Supplementary Material), the splenic CD4^+^ T cell number but not the frequencies were significantly elevated (Figure 4D; Figure S2C in Supplementary Material). Treg frequencies were significantly reduced in decidual tissue and Treg cell numbers were significantly diminished in spleens from NP female recipients of PC1^low^ cells (Figure 4C; Figure S2D in Supplementary Material), while changes in the splenic Treg frequencies did not reach statistical significance (Figure 4E). Moreover, the presence of PC1^low^ cells resulted in significantly elevated numbers of CD4^+^IFN-γ^+^ T cells as well as significantly increased frequencies and cell numbers of CD4^+^TNF-α^+^ and CD4^+^IL-17^+^ T cells in spleens (Figures 4F–H; Figures S2E–G in Supplementary Material). By determining the ratio between Treg and TH17 cells in the spleen, we found a significant reduced Treg/TH17 ratio after transfer of PC1^low^ cells (Figure 4I). By contrast, no changes in TH1 and TH17 frequencies and cell numbers were detected between both groups in the decidua (data not shown). In agreement with these data, mRNA expression analyses of IFN-γ and TNF-α at the fetal–maternal interface revealed comparable levels between the experimental and the control groups (Figures S3A,B in Supplementary Material). In addition, adoptive transfers of PC1^low^ B1a B cells did not influence the expression at the transcript level of the anti-inflammatory cytokines IL-10 and TGF-β (Figures S3C,D in Supplementary Material).

### Adoptive Transfer of PC1^high^ B1a B Cells Into AP Females Rescued Fetuses From Rejection

**Figure 5:**
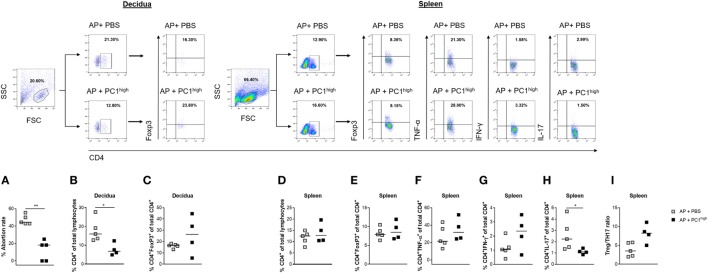
Adoptive transfer of PC1^high^ B1a B cells to AP females protected fetuses from rejection and induced changes in the TH compartment. 1 × 10^5^ peritoneal sort-purified PC1^high^ B1a B cells from normal pregnant females [gestation days (gds) 13–14] were adoptively transferred into AP females (*n* = 5) on gd7–9. Control females (*n* = 5) received PBS. The recipients were sacrificed on gd12 and the abortion rate **(A)** was determined. Moreover, CD4^+^ T cell **(B)** and CD4^+^Foxp3^+^ regulatory T (Treg) frequencies **(C)** were assessed in decidual tissue. In spleen, the frequencies of CD4^+^ T cells **(D)**, CD4^+^Foxp3^+^ Treg cells **(E)**, CD4^+^TNF-α^+^ **(F)**, and CD4^+^IFN-γ^+^ **(G)** TH1 cells as well as the frequencies of CD4^+^IL-17^+^ TH17 **(H)** cells and the Treg/TH17 ratio **(I)** were determined. Flow cytometry gating strategy and representative dot plots are included in this figure. Graphical data are displayed as medians. Each symbol represents a single animal, and the presented number of mice refers to pooled data of two independent experiments. Statistical analysis was performed using the non-parametric Mann–Whitney-*U* test (*p ≤ 0.05 and **p ≤ 0.01). Abbreviation: AP, abortion prone.

Next, we assessed whether the adoptive transfer of sorted PC1^high^ cells into the peritoneum of AP females was functionally relevant to rescue from abortion. Indeed, the transfer of PC1^high^ B1a B cells resulted in a significant reduced abortion rate (Figure 5A).

### The Protective Effect of PC1^high^ B1a B Cells Was Associated With Significantly Reduced Frequencies of Splenic TH17 Cells

To evaluate possible effects of the B cell transfer on TH cells, we determined the peripheral and local frequencies as well as cell numbers of total CD4^+^ T cells, Treg cells, TH1 cells, and TH17 cells. The protective effect of transferred PC1^high^ B1a B cells was associated with significant reductions in the frequencies and cell numbers of CD4^+^ T cells but not Treg cells in the decidua (Figures 5B,C; Figure S2I in Supplementary Material), while no changes were observed in spleens (Figure 5D; Figure S2K in Supplementary Material). In addition, while the presence of PC1^high^ cells had no significant effect on the frequencies and cell numbers of Treg cells, CD4^+^TNF-α^+^ and CD4^+^IFN-γ^+^ T cells in the spleen (Figures 5E–G; Figures S2L–O in Supplementary Material), there was a significant decrease in the frequencies of CD4^+^IL-17^+^ T cells in the same tissue (Figure 5H). The splenic Treg/TH17 ratio was, although not significantly, elevated in the presence of PC1^high^ cells (Figure 5I; Figure S2P in Supplementary Material).

Adoptive transfer of PC1^high^ cells into AP females was not associated with alterations in the frequencies and cell numbers of TH1 or TH17 cells at the fetal–maternal interface (data not shown). In keeping with these results, we did not detect significant differences in the mRNA expression levels of IFN-γ and TNF-α in the decidua, although TNF-α was modestly elevated in the B cell-transferred group (Figures S4A,B in Supplementary Material). Moreover, IL-10 and TGF-β transcript levels were not significantly altered in the experimental group, although TGF-β transcripts were slightly increased (Figures S4C,D in Supplementary Material).

## Discussion

Pregnancy-driven immunological adaptions resulting in a fine balance between pro- and anti-inflammatory immune responses are fundamental for the protection of the semi-allogeneic fetus in the mother’s womb. During the last several decades, many studies have explored the involvement of regulatory immune cell populations in fetal tolerance induction, the majority of them primarily focusing on Treg cells [reviewed in Ref. (13)]. Recent studies have highlighted the pivotal role for B cells, and particularly of Breg cells, for pregnancy success (1). Previous human and murine studies reported alterations in the frequencies of different B cell subpopulations during pregnancy progression and proposed an association with fetal tolerance (14–17). It has also been suggested that the nature of antibodies secreted by B1 and B2 B cells may determine fetal fate (5, 18, 19). Poly-reactive natural autoantibodies as well as anti-paternal alloantibodies have been shown to drive pregnancy complications (5, 18) whereas asymmetric “blocking” antibodies contribute to fetal survival (19).

Within the B1 B cell population, B1a B cells are of particular interest as these cells have been suggested to either protect or harm the fetus (5, 8). We previously reported that the anti-inflammatory cytokine IL-10 is critically involved in B1a B cell-mediated fetal tolerance induction (8) and the importance of IL-10 for normal pregnancy progression was further confirmed in human and murine studies (20, 21).

Hence, here, we first determined the frequencies and cell numbers of total B cells with the ability to produce IL-10 *in vitro* in NP females on different pregnancy days. While the frequencies and cell numbers of splenic CD19^+^IL-10^+^ B cells did not significantly change throughout pregnancy, the frequencies and cell numbers of peritoneal CD19^+^IL-10^+^ B cells increased significantly in the pre-implantation phase and declined thereafter. Notably, on gd10, the frequencies and cell numbers of peritoneal CD19^+^IL-10^+^ B cells were significantly decreased in AP females when compared with NP females, suggesting that IL-10 production by peritoneal B cells at mid-gestation is critical for fetal survival. However, we have to admit that the determination of IL-10 production in B cells after *in vitro* stimulation might not fully reflect the capability of the B cells to produce IL-10 *in vivo*. IL-10 reporter mice would be a powerful tool to further study the distribution of IL-10-producing B cells during normal and pathologic pregnancies in the future.

Next, we asked if the observed alterations in the peritoneal CD19^+^IL-10^+^ B cell pool are reflected within the B1a B cell subpopulation. It is noteworthy that Wang and colleagues described in non-pregnant animals two functionally distinct subsets of peritoneal B1a B cells (10) that may explain the dichotomy of this B cell subpopulation during pregnancy. The authors showed that the expression of PC1, a type II transmembrane glycoprotein, strongly correlates with the differential ability of the two B1a B cell subsets to secrete IL-10 (10). We observed a similar distribution pattern of peritoneal IL-10- and PC1-coexpressing B1a B cells throughout normal pregnancy to the total CD19^+^IL-10^+^ B cell population. Furthermore, on gd14, we detected lower frequencies and cell numbers of peritoneal IL-10- and PC1-coexpressing B1a B cells as compared with the normal pregnancy situation indicating that a lack of this specific B1a B cell subset coexpressing IL-10 and PC1 is associated with fetal rejection. However, as the numbers of peritoneal IL-10- and PC1-coexpressing B1a B cells are rather low and because less than 20% of the peritoneal CD19^+^IL-10^+^ B cells express CD5 our conclusions about the total peritoneal CD19^+^IL-10^+^ B cell pool may also apply to other IL-10-producing B cells.

In agreement with an earlier study (10), we confirmed that peritoneal PC1^high^ cells secreted more IL-10 than their PC1^low^ counterparts. In addition, PC1^high^ cells obtained from NP females secreted significantly more IL-10 than PC1^low^ cells from the same animals and more IL-10 than PC1^high^ B1a B cells from virgin females. This indicates that normal pregnancy boosts the ability of B cells to secrete IL-10 as it does with other cells, including T cells. For future studies, it would be interesting to analyze the pregnancy-specific signals that are involved in IL-10 secretion. We expect hormones to be one main stimulator (6). Interestingly, there were no detectable differences in the IL-10 secretory capacities of PC1^high^ and PC1^low^ cells obtained from the peritoneum of AP females. This reflects a failure of abortion-derived PC1^high^ cells to secrete more IL-10 in the face of high PC1 expression. The impaired ability of PC1^high^ cells from AP animals to secrete IL-10 might be associated with a reduced tolerogenic potential. Together with our finding that AP females showed a disturbed PC1^high^/PC1^low^ ratio on gd10, we postulate that an adequate balance between functionally competent (defined by IL-10 secretion) peritoneal PC1^high^ cells and PC1^low^ cells is a prerequisite for successful pregnancy outcome.

We performed adoptive transfer experiments to test our hypothesis that PC1^high^ cells are pregnancy protective because of their ability to secrete IL-10 while PC1^low^ cells are rather pregnancy destructive. We first adoptively transferred PC1^low^ cells to the peritoneum of NP females after implantation. This resulted in a harmful effect of PC1^low^ cells on fetal survival as evidenced by an increased fetal rejection rate. Transfer of PC1^low^ cells also provoked a reduction in the frequencies of decidual Treg cells and increased frequencies and cell numbers of splenic TH1. Notably, the splenic Treg/TH17 ratio was diminished after transfer of PC1^low^ cells. However, the local expression profile of the anti-inflammatory cytokines IL-10 and TGF-β as well as of the pro-inflammatory cytokines TNF-α and IFN-γ was not significantly affected.

By contrast, the adoptive transfer of PC1^high^ B1a B cells with the ability to secrete IL-10, to AP females significantly reduced their abortion rate and confirmed the substantial immune regulatory potential of this unique B1a B cell subset. In addition to the positive effect on pregnancy outcome after transferring PC1^high^ cells, we registered a statistically significant decrease in the frequencies and cell numbers of CD4^+^ T cells directly at the fetal–maternal interface. Furthermore, splenic TH17 cell frequencies were significantly decreased while splenic TH1 cells were not significantly affected. In keeping with our findings after transfer of PC1^low^ cells, we were not able to detect significant changes in the mRNA expression profiles of IL-10, TGF-β, TNF-α, and IFN-γ in decidual tissue. This implies that both B1a B cell subsets did not determine fetal fate primarily by affecting the local cytokine milieu. The most obvious changes induced by the transfer of both B1a B cell subsets were associated with changes in the splenic Treg and TH17 cell pools. Imbalances in the local and peripheral Treg/TH17 ratio are reportedly associated with several pregnancy complications such as unexplained recurrent pregnancy loss, preeclampsia and preterm birth [reviewed in Ref. (22)]. Direct and indirect mechanisms mediated by PC1^high^ and PC1^low^ cells may account for changes in Treg and TH17 cell numbers. On the one hand, soluble mediators, such as IL-10 or TGF-β, may provoke changes in the frequencies of Treg and TH17 cells (23). On the other hand, surface molecules expressed by B cells such as PD-L1 (24), Fas ligand (25), and Tim-3 (26) were implicated in affecting Treg and TH17 frequencies and thereby the ratio between the two T cell types. However, whether peritoneal PC1^high^ and PC1^low^ B1a B cells actually have the potential to influence Treg and TH17 frequencies or Treg/TH17 plasticity will require further studies. Other pathways involved in the differential effect of PC1^high^ and PC1^low^ cells on pregnancy outcome may include their differential effects on TH1 differentiation, the potential to secrete natural IgM antibodies, and/or their migratory activity (10). However, although the transfer of PC1^low^ cells had a greater impact on TH1 cells than the transfer of PC1^high^ cells, we do not believe that the differential regulation of TH1 responses by both B cell subsets is the reason behind the different effects on pregnancy outcome. Moreover, as we did neither evaluate the ability of the sorted peritoneal B1a B cell subsets to secrete natural IgM antibodies nor their migration capacity, this remains open to further study.

In conclusion, our former and present results suggest that B1a B cells play a major role for pregnancy success. Building on previous findings, we additionally propose that the peritoneum harbors two distinct B1a B cell subsets that can be defined by their expression of PC1 that also differ in their potential either to promote fetal tolerance or to induce fetal rejection. Thereby, the fine balance between tolerogenic PC^high^ and immunogenic PC1^low^ B1a B cells seems to be pivotal for pregnancy outcome.

## Ethics Statement

This study was carried out in accordance with the recommendations of the Guide for Care and Use of Animals in Agriculture Research and Teaching. The protocol was approved by the Landesverwaltungsamt Sachsen-Anhalt (AZ42502-2-868 UNIMD).

## Author Contributions

AS, SE, MS, and RH performed and analyzed experiments. AS and AZ designed and supervised the experiments. AS prepared figures, interpreted data, and wrote the manuscript. HW and HM contributed with reagents. HW, HM, and AZ critically revised the manuscript.

## Conflict of Interest Statement

The authors declare that the research was conducted in the absence of any commercial or financial relationships that could be construed as a potential conflict of interest.
