# Supplementary figures and images for: Plasma Cell Alloantigen 1 and IL-10 Secretion Define Two Distinct Peritoneal B1a B Cell Subsets With Opposite Functions, PC1high Cells Being Protective and PC1low Cells Harmful for the Growing Fetus
Source: Front Immunol. 2018 May 15;9:1045. doi: 10.3389/fimmu.2018.01045 (PMC5962664; doi:10.3389/fimmu.2018.01045)

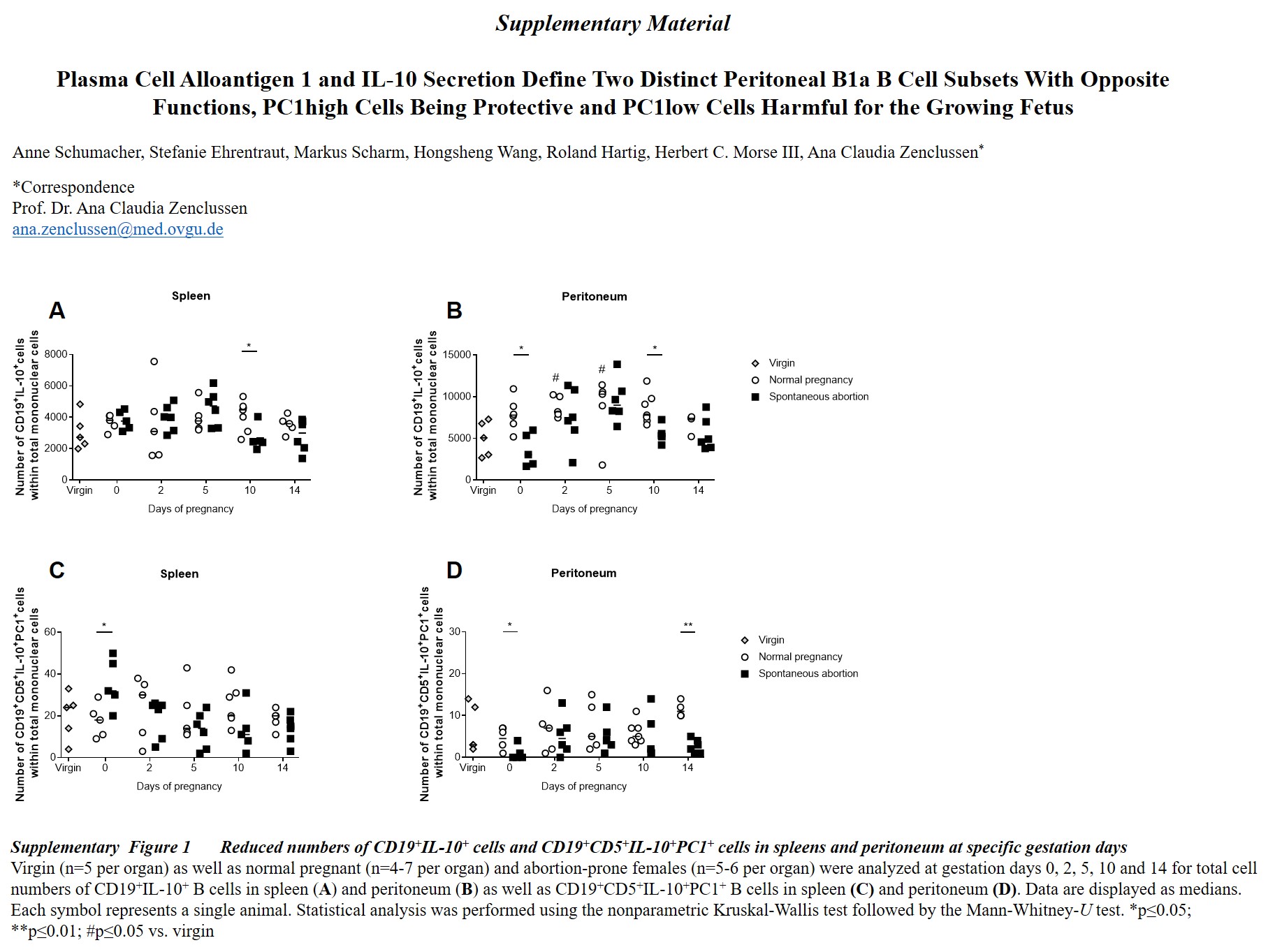

Supplement: Supplementary file 1 [file Image_1.jpg]

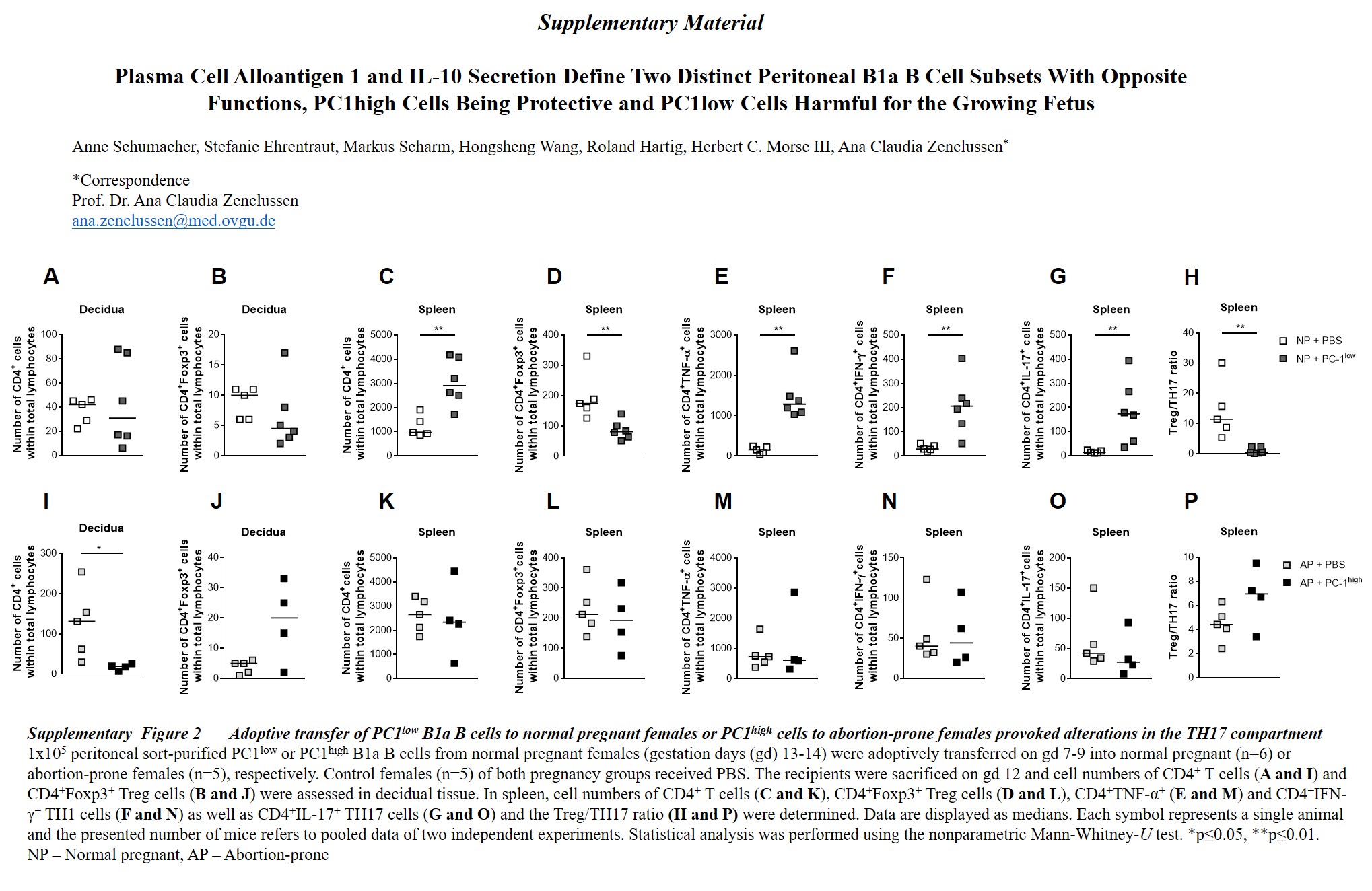

Supplement: Supplementary file 2 [file Image_2.jpg]

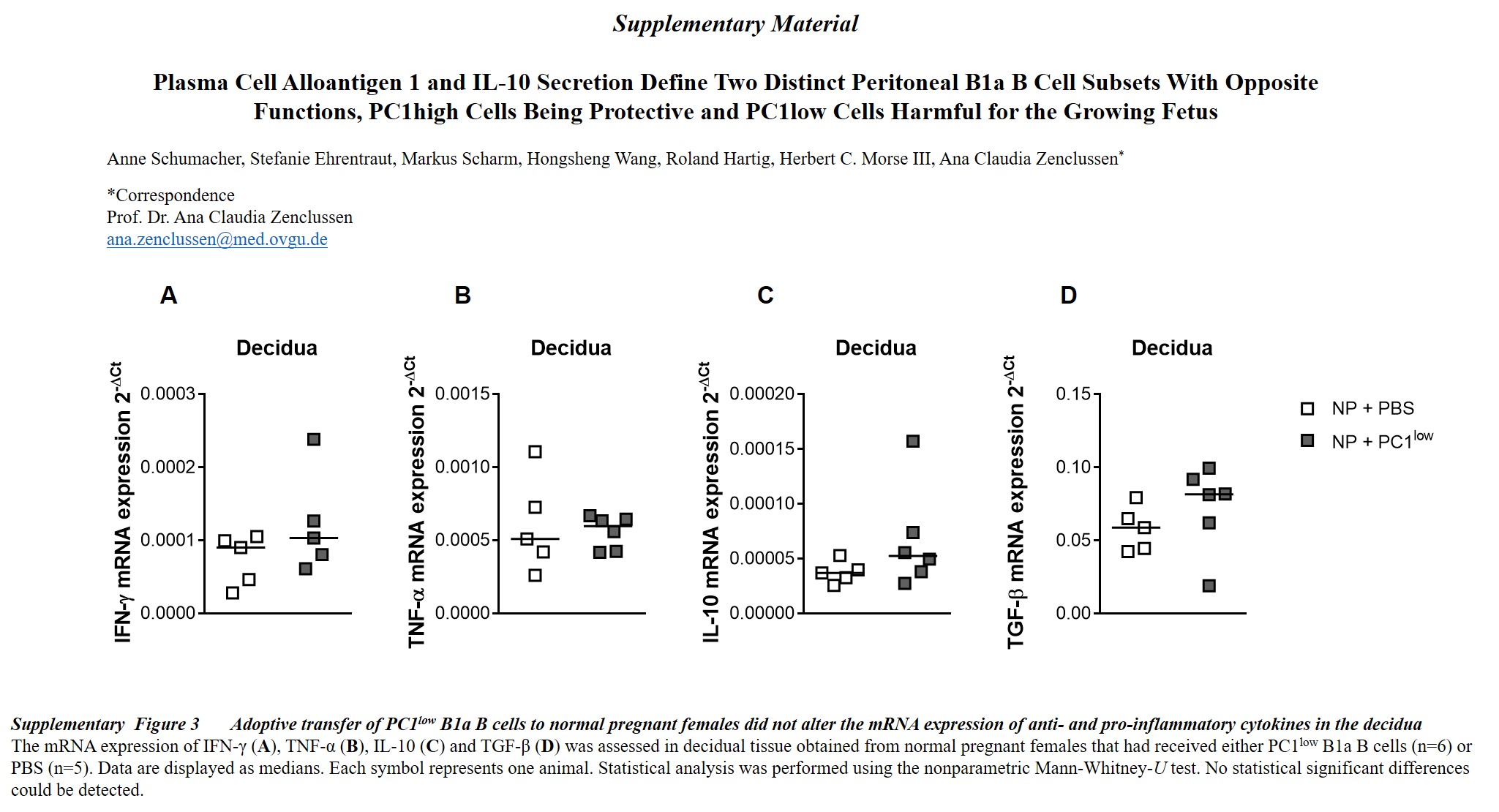

Supplement: Supplementary file 3 [file Image_3.jpg]

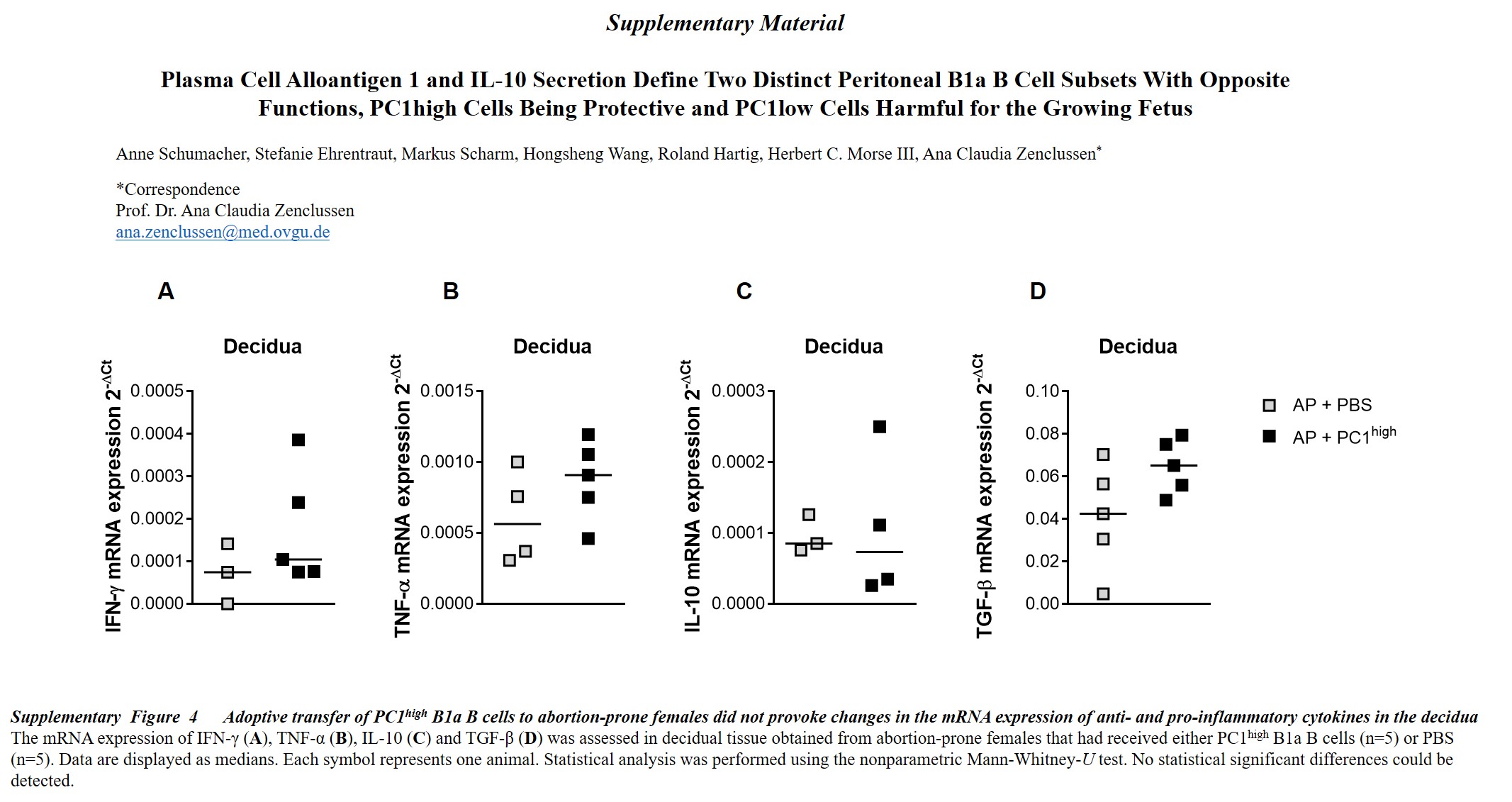

Supplement: Supplementary file 4 [file Image_4.jpg]
